# A Methodology for Designing Smart Urban Living Labs from the University for the Cities of the Future

**DOI:** 10.3390/s21206712

**Published:** 2021-10-09

**Authors:** Nadhiely Martínez-Bello, Mónica José Cruz-Prieto, David Güemes-Castorena, Alberto Mendoza-Domínguez

**Affiliations:** Tecnologico de Monterrey, School of Engineering and Sciences, Ave. Eugenio Garza Sada 2501, Monterrey 64849, Mexico; nadhiely.martinez@tec.mx (N.M.-B.); monica_cruzp@tec.mx (M.J.C.-P.); mendoza.alberto@tec.mx (A.M.-D.)

**Keywords:** smart cities, smart living labs, university of the future, off-grid building

## Abstract

Cities have high demand and limited availability of water and energy, so it is necessary to have adequate technologies to make efficient use of these resources and to be able to generate them. This research focuses on developing and executing a methodology for an urban living lab vocation identification for a new water and energy self-sufficient university building. The methods employed were constructing a technological roadmap to identify global trends and select the technologies and practices to be implemented in the building. Among the chosen technologies were those for capturing and using rain and residual water, the generation of solar energy, and water and energy generation and consumption monitoring. This building works as a living laboratory since the operation and monitoring generate knowledge and innovation through students and research groups that develop projects. The insights gained from this study may help other efforts to avoid pitfalls and better design smart living labs and off-grid buildings.

## 1. Introduction

Urban living labs (ULL) are spaces designed to facilitate experimentation about sustainability solutions. ULLs allow different urban actors to design, test, and learn from socio-technical innovations [1]; participation, experimentation, and learning are placed on center stage.

Universities can be used as a ULL since they function as a mini-city; many activities take place there, and, as a result, it is possible to generate models whose results can be extrapolated to cities. Reference [2] contributes to the city’s transformation into a smart sustainable city.

ULLs are spaces for collaborative experimentation of researchers, citizens, companies, and local governments; they provide a real-world testing ground for urban innovation and transformation [3]. Living labs are places to evaluate innovations in actual settings in highly visible ways, and they provide platforms for knowledge. They also help to attract attention and funding for new projects. These can be considered collaborative spaces between researchers, citizens, developers, and local authorities with shared objectives [3]. These labs can function as a mechanism for the design of the cities of the future. When we talk about universities, living labs are commonly focused on economic and sustainable urban development.

Besides testing technologies and processes under controlled conditions, living labs can showcase how the urban space could look, and also allow students to experiment in actual conditions through projects, challenges, or other educational schemes, as living labs work as a learning tool to enhance skill and advance research in several fields.

Currently, more than half of the population lives in cities, and this is highly likely to increase to more than two-thirds by 2030. Cities consume a large part of the world’s energy supply and are responsible for approximately 70% of global emissions of greenhouse gases [4].

The carbon footprint generated by cities is the result of poor planning and design. The dispersion of the suburbs with few means of public transport and homes far from work centers and commercial areas produces an increase in the circulation of cars emitting greater amounts of carbon dioxide, coupled with the fact that most buildings continue to use fossil sources to meet their energy needs [4].

Cities contribute directly to global warming, while they will be directly affected by extreme hydrometeorological events, scarcity of water resources, heat waves, poor air quality, and biodiversity loss [4].

According to the statement by the executive director of UN-Habitat, Maimunah Mohd Sharif [4], cities can be part of the solution to achieve a reduction in greenhouse gases that cause global warming, as they are also centers of innovation with the capabilities to generate technological and management solutions for energy, water, construction, and mobility sectors, and city planning, which has remarkable potential to reduce emissions significantly.

For this reason, it is imperative to generate solutions for the cities and from the cities that allow to reduce greenhouse gas emissions and contribute to the transformation of cities into smart sustainable cities [5] (p. 11):

“A Smart Sustainable City is a city that meets the needs of its present inhabitants without compromising the ability for other people or future generation to meet their needs, and thus, does not exceed local or planetary environmental limitations, and where this is supported by Information and Communication Technologies (ICT).”

This study aims to implement a ULL in a university through the E2 project, which seeks to be 100% self-sufficient for the water and energy necessary for the building’s operation.

This project aims to select different technologies and best practices to be implemented and assessed in a building, and the results are observed through constructing a technological roadmap. The data generated would allow university students and research groups to generate knowledge by developing projects.

## 2. Methods

In this section, we describe the following methods: (1) megatrend analysis, (2) technology roadmapping, and (3) technology evaluation and research capabilities mapping.

### 2.1. Megatrend Analysis

Megatrends have been described in classic books such as Megatrends 2000 by John Naisbitt and Patricia Aburdene [6] and Future Shock by Alvin Toffler [7]. Megatrends have been defined as the great forces in human and technology development that affect the future in many areas of human activity on a horizon of ten to fifteen years, according to Naisbitt and Aburdene. Although these books provide an excellent forecast about imminent socioeconomic trends, other authors provide methodologies to analyze megatrends and to identify opportunities from them [8,9,10].

This study used this analysis to identify those megatrends that matched the different stakeholders’ future expectations. Megatrend analysis was limited to the following specific areas: off-grid buildings, smart buildings, energy, water use, and education (since the case was applied to a university building).

The megatrend analysis was performed to provide the stakeholders with a holistic context and the possible role of the set of buildings within the regional context; also, the analysis helped to identify future scenarios that the decision-makers had not foreseen. The megatrends provided possible future scenarios, and the decision-makers could identify the characteristics of the new building to mitigate the city’s energy and water burden.

The megatrends were identified from diverse sources, including specialized reports on water and energy issues from international organizations such as The Organisation for Economic Co-operation and Development (OECD), consulting companies such as McKinsey and Company, and Frost & Sullivan, and journal papers studying these topics. For water megatrends, the sources were mainly the following [11,12,13,14,15,16]; regarding the energy megatrends, the sources were mainly from [16,17,18,19,20,21]; lastly, the sources for smart buildings and the future cities were from these sources [15,16,21,22,23,24,25].

The identification of the megatrends was based on the following criteria: (i) worldwide relevance, (ii) local applicability, and (iii) related to the water, energy, and the future of the cities. The trends that were initially identified are presented in Table 1.

Megatrends from Table 1 were analyzed to identify the needs and technologies that were forecasted. Table 2 exemplifies some categories for the water and energy-related megatrends, where the future needs were identified. The needs identification came from an abstract conceptualization of the triggers and detonators for each megatrend.

From the future needs, a range of technological options was identified. We utilized the Goldfire Innovator Software for the technology identification, where semantic searches were performed in journals, patents, and norms databases to cover all the scientific knowledge, to-be-commercialized technologies, and industry norms. The searches were performed to find what technologies were scientifically and technologically available. Goldfire Innovator Software offers a semantic search where it classifies the searches by applications and methods, among many other ways to refine the findings.

### 2.2. Technology Roadmaps

The construction of a technology roadmap was relevant since it is a planning tool that used the megatrends as the contexts and scenarios of how a region may develop, and the stakeholders decided which living labs could be set within the buildings, as well as the technologies that could be installed to provide the off-grid characteristics. Since the roadmap is a visualization of the future needs and the technologies that may satisfy those needs, selecting the technologies is the next step in the process.

### 2.3. Technology Evaluation and Research Capabilities Mapping

Technology evaluation was a necessary step in the process. Since many technologies are available and under development that may satisfy a future need, the university must select those technologies that may offer a competitive advantage. Therefore, a set of technology evaluation tools were designed to select the technologies with higher opportunities. These evaluation tools measured the technology attractiveness (see Table 3), the internal know-how (see Table 4), and the need for action (see Table 5).

Technology attractiveness evaluation relates the technology to market potential. It includes the evaluation of technological maturity and market possibilities. Table 3 provides the detailed rubric that was utilized for each technology under consideration.

The internal know-how evaluation quantifies the technological maturity of the organization; in the evaluation, the publication and people capabilities were measured as well as the number of projects developed by the university and the collaboration capabilities (see Table 4).

The internal need for action evaluation quantifies the urgency to develop the technology. It considers the following elements: capabilities availability, resources availability, customers, the technology-need-megatrend alignment, the regional importance of the technology, and the alignment to the university’s strategy (see Table 5).

Similarly, through a technology search, all the technologies were evaluated according to (1) their future market potential impact and (2) their technological complexity. These two elements were evaluated to identify, according to external experts, the social impact of the technology and how difficult it is to develop. The evaluation scale was performed using the following scale: 0—none, 1—very low, 2—low, 3—medium, 4—high, and 5—very high.

Once the individual technologies were evaluated, the process continued to select those with the greatest possibility of success by considering the most attractive technologies that can be developed within the university and with the appropriate need for action. This process reduces the number of technologies to a handful.

## 3. Results

This section describes the methodology results and the implementation of the results in the E2 building.

### 3.1. Megatrend Analysis Results

The stakeholders were shown all the megatrends and were asked to select one-third (or less) of all megatrends. All the stakeholders evaluated the megatrends, and some of them changed the focus and name slightly. The most important ones for the stakeholders are shown in Table 6; the importance was relative to the stakeholders´ objectives and their specific interests in this project.

The next step was to determine the “needs” that would trigger those megatrends. Uncovering the “needs” is critical in identifying the problems that may happen. These “needs” are shown in Table 7, showing those related to the sensing. The solution to those problems was the possible technologies that the smart building may incorporate or that the university may research. Technology scouting was needed to determine what technologies are focused on solving those needs; since there are many technologies to solve those problems, the authors focused on technologies that can be incorporated into buildings and have—or promise to have—higher efficiency than the commercial ones.

A variety of technological options were found based on future needs. Table 8 shows the thirteen technologies—and their descriptions—that could be the focus of the living lab. These technologies were selected by searching in Goldfire Innovator Software scientific journals databases, most patent offices´ databases, and industry norms databases for the technological solutions to those needs. According to the technology attractiveness, internal know-how, and the university´s need for action, a brief technology description was shared with the researchers for their evaluation.

### 3.2. Technology Roadmapping Results

**Figure 1:**
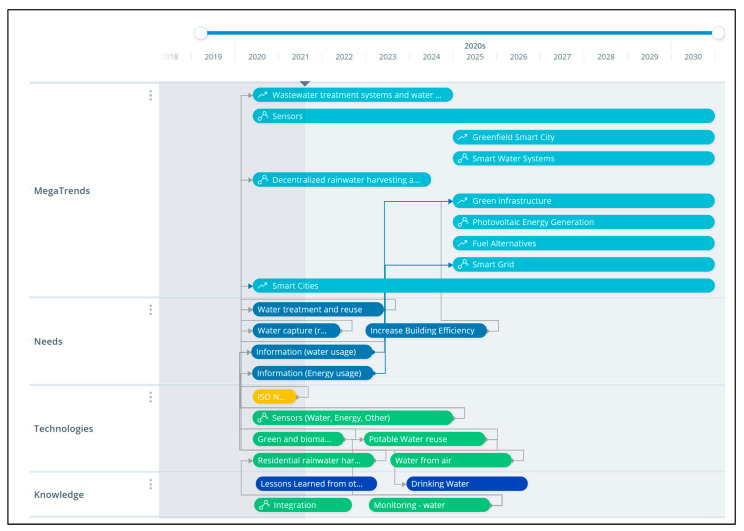
Megatrends, needs, and technologies identified and aligned.

A technology roadmap was created to represent the technologies, needs, and megatrends relationships and interconnections according to the selected megatrends. The technology roadmap is an excellent tool to visualize possible technology development options [26]. A representation of the roadmap can be seen in Figure 1, where a timeline helps locate the technology developments, their connection to the “need”, and their impact on the megatrend (see the interconnecting arrows.) The connection that a roadmap makes helps decision-makers visualize the impact a technology development could have in the future.

### 3.3. Technology Evaluation and Research Capabilities Mapping

Technology attractiveness evaluation is an exogenous evaluation. It measures the technology´s market potential worldwide. On the other hand, the internal know-how and the need for action evaluations measure the university´s capabilities. With these exogenous and endogenous evaluations, a better decision can be made.

Fifty-six questionnaires were sent out to professors to gather their perspectives on the different technologies’ characteristics: attractiveness, internal know-how, and need for action. Only twenty-eight responses were returned; at least one answer was received per technology, and a dispersion analysis was performed per technology—to ensure there were not highly contrasting perspectives on a technology, which there were not. We present, in the following figures, the results of each evaluation and its interpretation. Since the graphs were difficult to read with all the thirteen technologies, we simplified the graphs by eliminating some technologies that the stakeholders did not select.

As described in Section 2.3, the technologies were evaluated according to the exogenous factors (potential impact, complexity, and technology attractiveness.) Let us keep in mind that technological attractiveness was assessed by research professors, as well as an evaluation according to technology reports on the subject. In our case, both evaluations were the same for the technologies. A summary of the evaluations is presented in Table 9.

**Figure 2:**
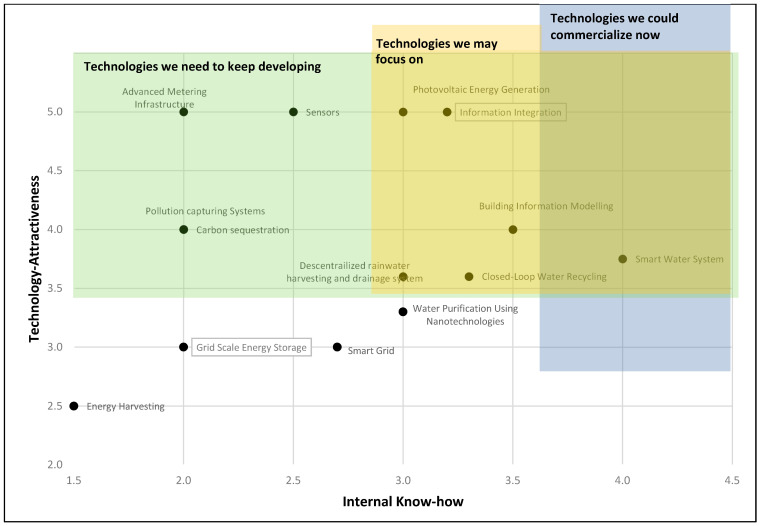
Technology attractiveness vs. internal know-how.

**Figure 3:**
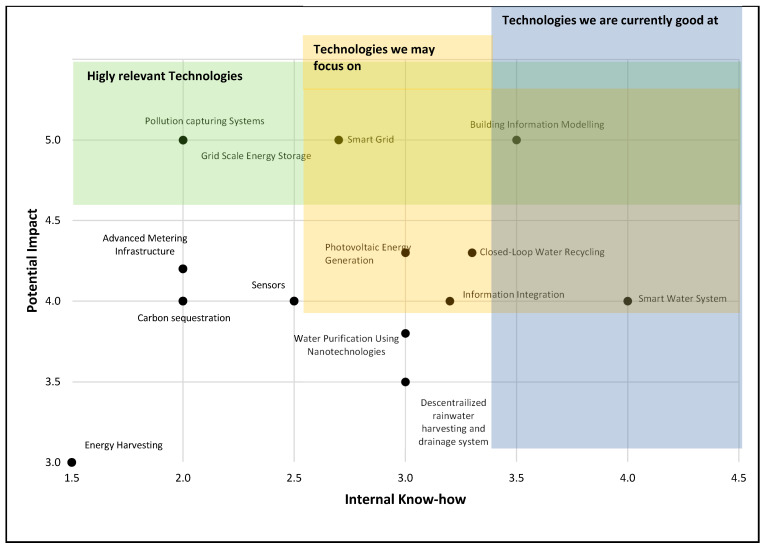
Potential impact vs. internal know-how.

**Figure 4:**
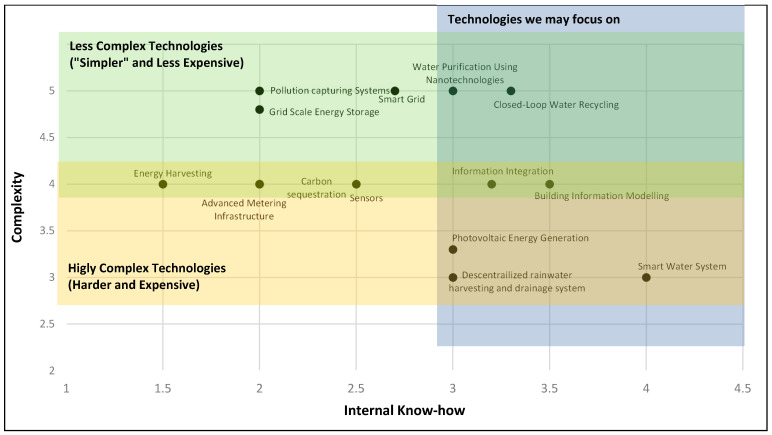
Complexity vs. internal know-how.

Information in Table 9 can be analyzed in diverse ways. We present three graphs that helped us select those relevant technologies for the university, the building, and the stakeholders in the project. The first analysis is shown in Figure 2; it presents the technology attractiveness vs. the technology’s internal know-how. As mentioned in Section 2.3, the scale is 0 to 5, where 5 is the highest value. Figure 2 has three shaded areas:

- Green area: this area represents those technologies with a high technology attractiveness value. It represents those technologies that the university may continue developing for their high attractiveness.
- Blue area: this area represents those technologies the university is developing with the highest internal know-how evaluation. Those technologies are the ones the university may commercialize or transfer in the short term because of their expertise. This area’s description is the same for Figure 3 and Figure 4.
- Yellow area: this area is intended to cover the most valuable intersection for the green and blue areas, highlighting the most important technologies to focus on, according to the technology attractiveness and internal know-how evaluation.

A similar analysis was performed using the technology’s market potential impact and internal know-how. This analysis is shown in Figure 3, and the shaded areas represent the following technologies:

- Green area: this area represents those technologies with a high technology impact value. It represents those technologies that the university may continue developing for their future high impact on the market.
- Blue area: this area represents the university’s technologies with the highest internal know-how evaluation, as described in Figure 2.
- Yellow area: this area is intended to cover the most valuable intersection for the green and blue areas, highlighting the most important technologies to focus on, according to the technology’s potential impact and internal know-how evaluation.

The third analysis is the technology’s complexity vs. internal know-how, as represented in Figure 4. The complexity of developing a specific technology is related to the resources needed to develop the science behind the technology, the time it takes to commercialize it, and the required infrastructure. This analysis is shown in Figure 4, and the shaded areas represent the following technologies:

- Green area: this area represents the top part of the region, where the technologies that fall within this section are “harder and more expensive” to develop.
- Yellow area: this area represents the bottom part, identifying the less complex technologies, “simpler and less expensive” to develop.
- Blue area: this area is on the right part of the graph, representing those technologies for which the university has the know-how, regardless of their complexity.

The selected technologies were those falling in the “technologies we may focus on” areas of Figure 2, Figure 3 and Figure 4 since they combine the internal technological know-how and the highest technological attractiveness and highest potential impact. Table 10 summarizes the graphical analysis in a table form; selection criteria I represents the technologies selected from Figure 2, selection criteria II represents the technologies selected from Figure 3, and selection criteria III represents the technologies selected from Figure 4. The selection highlights six technologies that comply with at least two selection criteria, and three of those technologies are water-related; these six technologies are shaded in Table 10.

The selection of these technologies that helped design the smart building and the ULL is explained in the following section.

### 3.4. Designing the Smart Living Lab

**Figure 5:**
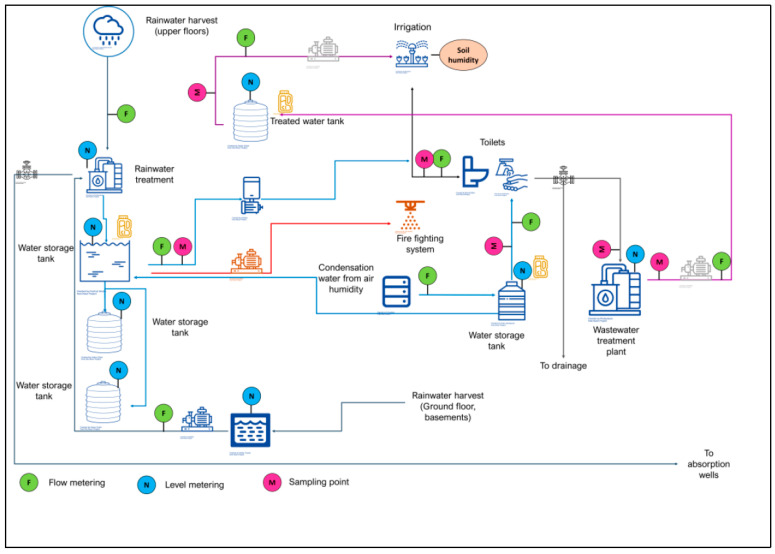
Water system flow chart.

The new building design incorporated water and energy technologies, which will render the building off-grid and provide a living lab to be used by the students. All the technologies installed were selected according to the results obtained from the technological roadmap, as all were following the megatrends to solve urban problems concerning off-grid water and energy supply. The instruments and the monitoring system installed were a fundamental part of this ULL since the academic community uses the information generated at the facility for projects and human capital training, as well as for a source of dissemination of data about consumption, self-sufficiency, and building experiences, which could be extrapolated to other buildings with similar characteristics. Besides, the building is part of the smart campus city project, which has been undergoing development. This section describes the proposed system for collecting rainwater and its sensing components, schemed in Figure 5.

Rainwater is collected mainly on the roof of the building’s top floor, which solar panels cover. The rainwater collection system goes down to the basement, where the rainwater treatment system is located, and its objective is to remove the pollutants, so after the treatment, the water quality is adequate to be used in toilets and building services. The treated rainwater goes then to storage tanks; from here, the water is used mainly for the building’s toilets, the fire-fighting system, and the green areas’ irrigation adjacent to the building.

An atmospheric humidity condensation system supplies the water for the sinks. Both the sinks’ and toilets’ gray water and wastewater are sent to a wastewater treatment plant, where it is treated to be reused to irrigate the green areas.

There were installed flow and level meters at various points connected to a central information system to continuously monitor the tanks’ levels and the flow rate treated and consumed. Energy meters were also installed to monitor the energy requirements of the water collection and treatment system and the rest of the building’s services. The energy generated by the solar modules was installed on the roof, and it is being monitored too, to determine if, at the year’s end, the building has reached net-zero in water and energy.

Regarding the power generation system, as mentioned above, the building has solar modules throughout the roof area that generate the necessary energy to cover 100% of the building’s energy requirements, including the consumption of three commercial spaces located in the front of the building. Energy meters for the overall consumption of the building were also installed to monitor its energy production and consumption.

## 4. Discussion and Conclusions

This study developed a methodology to help identify the technologies to be installed and researched in a university setting as part of the core technologies for a ULL.

The technology identification process was screening, through different criteria, the water and energy-related technologies to be installed in a net-zero building. Those technologies were considered a priority since the building location is in a water stress area. Furthermore, the region has been going through a prolonged drought period. This paper sets a methodology for designing smart buildings and ULLs by helping different stakeholders agree on the core technologies for the ULL; those technologies have been selected since they increase the chances of contributing to society and differentiate the university’s research capabilities.

The selected technologies for the case study:

- Rainwater collection and treatment systems to discharge into toilets and general building services.
- Generation of water from the condensation of humidity in the air for sinks and drinking fountains.
- Treatment plant for wastewater and greywater, whose discharge will be reused in the irrigation of green areas.
- A solar photovoltaic generation system, installed on the roof of the building, which also collects rainwater.
- A monitoring system for water and energy generation and consumption.

The methodology has helped to reach several objectives for the ULL. From the academic perspective, the building works as a ULL and contributes to the student learning process by developing projects in a real environment. In addition, this building has helped advance disciplines directly related to engineering, public policy, and urban planning. This facility also brings in additional research funds and enables the university to qualify for new grants and certifications in the future. By functioning as a smart ULL, this building contributes to facilitating the city’s transformation into a sustainable smart city and making society aware of the efficient use of resources in an urban environment.

This work was focused on identifying technologies that directly affect the essential operation of the building (water and energy) since the budget was limited. There are still many other technologies that were very close to being implemented. However, for the next stage, the project planners consider the technologies that came from the technology roadmap as a priority, such as CO_2_ capture and energy generation by non-conventional means (photovoltaic glasses, piezoelectric tiles).

The findings of this research have several important implications for future practice. The insights gained from this study may help other efforts to avoid pitfalls and better design smart ULLs. Most ULLs and net-zero buildings are designed and installed in developed countries; this methodology and case application can help universities identify and strengthen their core research competencies by designing ULLs for their universities to change their regions’ mindset exponentially.

## Figures and Tables

**Table 1:** Water, energy, and future of the cities´ megatrends.

Alternative Energy Adoption	Green Supply Chain	Smart Cities
Closed-Loop Water Recycling	Mega Cities	Smart Lighting
Connected Mobility	Microgrids	Smart Public Services
Consumer Habits Regarding Water Use	Micro Mobility	Wastewater Treatment Systems and Water Reuse
Fuel Alternatives	Natural Resources Stress	Water Allocation and Distribution
Green Infrastructure	New Business Models with Water Infrastructure Investment	Water as a Luxury
Green Manufacturing Growth	Off-Grid Energy	Water Deficit
Greenfield Smart City Infrastructure	Pollution Capturing System	Water Purification Using Nanotechnologies
Hyper Growth Energy Demand	Rewarding Sustainable Behavior	Zero Waste

**Table 2:** Exemplification of the relationship between a megatrend and future needs.

Megatrend	Future Needs
Water-related megatrends	Better water collection
	Methods to purify water
	Rainwater collection and treatment
Energy-related megatrends	Monitoring/measurement
	Improve energy efficiency

**Table 3:** Technology attractiveness evaluation.

Evaluation → Technology Attractiveness ↓	None	Very Low	Low	Medium	High	Very High
	0	1	2	3	4	5
**Market Potential**	No clear application No clear industry that may adopt it	A possible application No clear industry that may adopt it	A possible application 1 industry may adopt it	Some applications 1 industry may adopt it	Some applications Many industries may adopt it	Many applications Many industries may adopt it
**Competitive Situation**	Research papers without a specific application No patents yet	Unknown competitors. No patents yet	Unknown to few competitors. Few patents under development	5 possible competitors Few published patents	5–15 competitors Some published patents	20+ competitors Many published patents
**Technology Potential**	Theory under development	Theory under development Expected improvement unknown	It is based on a solid theory. Expected improvements are known	May substitute a technology. Improvements in 3X	Will substitute a technology. Improvements in 10X	Will substitute other technology. Improvements in 100X

**Table 4:** Internal know-how evaluation.

Evaluation → Demonstrated Capabilities ↓	None	Very Low	Low	Medium	High	Very High
	0	1	2	3	4	5
**Publication capabilities**	Neither Conference nor journal papers	Conference +(3–6) journal papers	Conference + (6–10) journal papers	(10–20) journal papers	(20–40) journal papers per year	(40+) journal papers per year
**People capabilities**	No one is working on this topic No graduate degree course on this topic	At least 1 person on this topic An optative graduate degree course on this topic	At least 2 people on this topic at the Tec of MTY An optative graduate degree course on this topic	At least 3 people on this topic at the Tec of MTY A graduate degree course on this topic	Research chair with at least 3 people on this topic A master´s degree on this topic (alignment)	Research chair with at least 5 people on this topic A Ph.D. + master´s degree on this topic (alignment)
**Number of projects**	No projects on this topic	One project under development with this technology	One project under development with this technology	Projects (1–3) developed with this technology	Projects (5–10) developed with this technology	Projects (10+) developed with this technology
**Collaboration capabilities**	No collaboration network yet	Limited collaboration network	National collaboration network	National collaboration network	International collaboration network	International collaboration network

**Table 5:** Need for action evaluation.

Evaluation → Need for Action ↓	None	Very Low	Low	Medium	High	Very High
	0	1	2	3	4	5
**Technological capabilities availability**	We do not have the capabilities	We have few scattered capabilities, and no plan to build more	We have few capabilities, and we are building more	We have some capabilities, and we are building more	We have most of the capabilities	We have all the capabilities
**Resources availability (financial + labs + partners)**	We do not have the resources	We have few resources, and no plan to build more	We have few resources, and we are building/accessing more	We have some resources, and we are building/accessing more	We have most of the resources	We have all the resources
**Customers for the technology**	We have no customer on sight	We have no customer on sight	We have one customer	We have few customers	We have some customers	We have many customers
**Technology, need, and megatrend alignment**	The technology is not aligned to a megatrend	The technology is not aligned to a megatrend	The technology is not clearly aligned to a megatrend	The technology is semi-aligned to a megatrend, and the market potential is high	The technology is semi-aligned to a megatrend, and the market potential is high	The technology is aligned to a megatrend, and the market potential is very high
**Regional importance**	It is not an important issue	It is an important issue for a company	It is an important issue for a region in Mexico	It is an important issue for Mexico	It is an important issue for Mexico or the world	It is an important issue for Mexico and the world
**Alignment to the strategy of the University**	It is a technology not necessarily aligned to the university	It is a technology for a specific project at the University	It is an important technology for a specific project at the University	It is an important technology for some projects at the University	It is an important technology to achieve the mission of the University	It is a very important technology to achieve the mission of the university

**Table 6:** Selection of the relevant megatrends.

Sensors	Greenfield smart city infrastructure
Smart grid	Green infrastructure
Smart cities	Photovoltaic energy generation
Fuel alternatives	Wastewater treatment systems and water reuse
Smart water systems	Decentralized rainwater harvesting and drainage systems

**Table 7:** The identified water and energy “needs” from megatrends.

Water	Collection	
	Purification	
	Rainwater collection and treatment	
	Monitoring/measurement	↔ Sensing
	Efficiency	
	Wastewater treatment	
	Treatment and reuse	
Energy	Generation Efficiency	
	Monitoring/measurement	↔ Sensing

**Table 8:** The selected technologies with a brief description.

Advanced Metering Infrastructure	Allows two-way data transmission between the customer and the utility. As well as being a tool for the active influence of customer behavior, it can detect leaks at the individual household level and detect anomalous usage patterns.
Building Information Modelling	It is a system that involves generating and managing digital representations of places’ physical and functional properties. It provides an intelligent 3D model of an area that gives insights and tools to plan, design, construct, and manage buildings more efficiently. BIM creates a unique perspective of the building process, saving money and time, and simplifying the entire construction development procedure.
Carbon Sequestration	CO_2_ is the most produced greenhouse gas and a major cause of global warming. Carbon sequestration occurs both naturally and as a consequence of anthropogenic activities. Success in this area will benefit the environment and industries such as oil and gas, agriculture, renewable energy, and industrial construction.
Closed-Loop Water Recycling	The process restores and regenerates waste as part of its design to keep materials at their highest utility and value. The process will help wastewater producers reduce their carbon footprint and achieve corporate sustainability
Decentralized Rainwater Harvesting and Drainage System	Decentralized systems also apply to stormwater drainage, with a growing use of “source control” technologies that handle stormwater near the point of generation, i.e., locally. For instance, green roofs or pervious surfaces capture rainwater before it runs onto polluted pavements and streets.
Energy Harvesting	Energy harvesting is the process of pulling ambient energy from external environmental sources. The energy, once captured, is stored to be used. Currently, it is applied to areas such as building and industry automation, smart cities, automotive vehicles, and security systems, for example. Developments in big data, the Internet of Things, and the need to replace batteries, are major forces driving advances in energy harvesting technologies.
Grid-Scale Energy Storage	Storing energy in this way provides the needed flexibility to manage dynamic resources effectively while reducing the overall costs associated with peak-time energy transmission. Most new grid-scale energy storage solutions are based on lithium-ion battery technology. However, research reveals alternative solutions, such as liquid silicone storage, which may economically secure energy in a greater capacity.
Information Integration	Networking technology connects all kinds of equipment and machines to integrate related virtual and physical services.
Photovoltaic Energy Generation	As photovoltaic technologies develop, they are offering higher conversion efficiency and lower costs to traditional solar panels. Photovoltaics appeal to the construction industry as the panels aid in achieving energy targets for building design. At the same time, the renewable energy sector will benefit through the offering of better energy performance.
Pollution-Capturing Systems	Pollution-capturing systems or smog-eating technology refers to newly produced materials designed to capture toxic air pollution and hold it within the core of the material, preventing it from escaping. These come in the form of CO_2_-infused concrete, smog-eating tiles, and large city air purifiers. These materials and systems are increasingly being tested and applied to construction projects, contributing to the improvement of the environment and reducing overall construction costs.
Sensors	Water, power, and energy monitoring: A new type of digital water and power meter can accurately indicate the amount of energy consumption and report the information over the network. This helps to increase efficiency and helps to ensure the best use of the resources. The employment of sensors along the transmission route creates the recording of vital information that can then be relayed.
Smart Grid	A smart grid refers to digital technology enabling two-way communication between an energy utility and its customers. Smart grids allow for real-time electricity supply and demand data collection, simultaneously alongside transmission and distribution. Real-time data collection makes evaluation, monitoring, consumption, and maintenance much more efficient.
Smart Water System and Water Purification using Nanotechnologies	This refers to the use of various nano-water filtration systems and techniques to purify all contaminated water types. Nanotechnologies are viewed in science circles as a viable solution to the problem due to manipulating core properties at the nano level while being cost-efficient.

**Table 9:** Need for action evaluation.

	Evaluations →	Potential Impact	Complexity	Technology Attractiveness	Internal Know-How	Need for Action	TRL ^1^
Technologies ↓							
Advanced Metering Infrastructure		4.2	4	5	2	2	9
Building Information Modelling		5	4	4	3.5	3.5	7
Carbon sequestration		4	4	4	2	3	3
Closed-Loop Water Recycling		4.3	5	3.6	3.3	4	3
Decentralized Rainwater Harvesting and Drainage System		3.5	3	3.6	3	3	9
Energy Harvesting		3	4	2.5	1.5	2	7
Grid Scale Energy Storage		5	4.8	3	2	3	7
Information Integration		4	4	5	3.2	3.3	7
Photovoltaic Energy Generation		4.3	3.3	5	3	3	9
Pollution-Capturing Systems		5	5	4	2	3	7
Sensors		4	4	5	2.5	2.5	9
Smart Grid		5	5	3	2.7	4	7
Smart Water System		4	3	3.75	4	4	8
Water Purification Using Nanotechnologies		3.8	5	3.3	3	3	8

^1^ Technology readiness level.

**Table 10:** Technology evaluation criteria.

Technology ↓ Selection Criteria →	I	II	III
Advanced Metering Infrastructure			
Building Information Modelling	√	√	√
Carbon Sequestration			
Closed-Loop Water Recycling	√	√	√
Decentralized Rainwater Harvesting and Drainage System	√		√
Energy Harvesting			
Grid-Scale Energy Storage			
Information Integration	√	√	√
Photovoltaic Energy Generation	√	√	√
Pollution-Capturing Systems			
Sensors			
Smart Grid		√	
Smart Water System, Water Purification, and Recycling	√	√	√
